# The development of HIV vaccines targeting gp41 membrane-proximal external region (MPER): challenges and prospects

**DOI:** 10.1007/s13238-018-0534-7

**Published:** 2018-04-17

**Authors:** Huan Liu, Xiaojie Su, Lulu Si, Lu Lu, Shibo Jiang

**Affiliations:** 1 State Key Laboratory of Virology, Wuhan Institute of Virology, Chinese Academy of Sciences, Wuhan, 430071 China; 2 Key Laboratory of Medical Molecular Virology of MOE/MOH, School of Basic Medical Sciences & Shanghai Public Health Clinical Center, Fudan University, Shanghai, 200032 China; 3 New York Blood Center, Lindsley F. Kimball Research Institute, New York, NY 10065 USA

**Keywords:** HIV-1, gp41, MPER, vaccine, neutralizing antibodies, ADCC

## Abstract

A human immunodeficiency virus type-1 (HIV-1) vaccine which is able to effectively prevent infection would be the most powerful method of extinguishing pandemic of the acquired immunodeficiency syndrome (AIDS). Yet, achieving such vaccine remains great challenges. The membrane-proximal external region (MPER) is a highly conserved region of the envelope glycoprotein (Env) gp41 subunit near the viral envelope surface, and it plays a key role in membrane fusion. It is also the target of some reported broadly neutralizing antibodies (bNAbs). Thus, MPER is deemed to be one of the most attractive vaccine targets. However, no one can induce these bNAbs by immunization with immunogens containing the MPER sequence(s). The few attempts at developing a vaccine have only resulted in the induction of neutralizing antibodies with quite low potency and limited breadth. Thus far, vaccine failure can be attributed to various characteristics of MPER, such as those involving structure and immunology; therefore, we will focus on these and review the recent progress in the field from the following perspectives: (1) MPER structure and its role in membrane fusion, (2) the epitopes and neutralization mechanisms of MPER-specific bNAbs, as well as the limitations in eliciting neutralizing antibodies, and (3) different strategies for MPER vaccine design and current harvests.

## Introduction

Acquired immunodeficiency syndrome (AIDS) is an infectious disease caused by human immunodeficiency virus (HIV) infection, which can impair and even destroy the human immune system. Since its discovery in 1983, AIDS has spread worldwide with more than 36.7 million people who are living with HIV infection, thus calling for development of effective and safe vaccines to prevent HIV infection and end the current AIDS pandemic. The statistical analysis based on a mathematical model predicts that application of a 50%-efficacy vaccine starting from 2020 and gradually scaling up to 70% coverage by 2035 will avert 17 million new infections if the current conditions of diagnosis and treatment keep unchanged (Medlock et al., 2017).

In spite of efforts for more than 30 years and hundreds of clinical trials, most HIV vaccine clinical trials have failed and none of the HIV vaccines has been approved so far. The RV144 vaccine trial that was launched in Thailand in 2009 is the only clinical trial showing an efficacy of 31.2% reduction of HIV type 1 (HIV-1) infection (Kim et al., 2015), and since then, no more effective HIV-1 vaccine has been developed. However, several groups have discovered some vaccine targets on the virus surface which play an important role in the infection process, such as the CD4 binding site (Wu et al., 2009), V1V2 region (Wang et al., 2017) and membrane-proximal external region (MPER: _659_ELLELDKWASLWNWFDITNW LWYIK_683_, HXB2 numbering) (Sun et al., 2016). All these targets are located on the HIV-1 Env. Eliciting broadly neutralizing antibodies (bNAbs) against these targets, i.e., antibodies that can neutralize a broad spectrum of HIV-1 strains, is one of major goals for designing a successful HIV-1 vaccine (Haynes and Mascola, 2017).

MPER is a highly conserved motif in the HIV-1 Env gp41 subunit near the viral envelope surface. It plays an important role in membrane fusion, and it is the target of some reported bNAbs. Thus, MPER is deemed to be one of the most promising vaccine targets. Multiple monoclonal antibodies (mAbs) against this region have been reported so far, such as 2F5, 4E10, Z13, Z13e1, m66.6, CH12 and 10E8 (Muster et al., 1993; Muster et al., 1994; Stiegler et al., 2001; Zwick et al., 2001; Nelson et al., 2007; Hessell et al., 2010; Morris et al., 2011; Huang et al., 2012; Ofek et al., 2014). Among these antibodies, 2F5, 4E10 and 10E8 exhibit broadly neutralizing activity, but these kinds of bNAbs cannot be elicited in animals through immunization. Many attempts have been made to develop vaccines targeting MPER, but only a small number of them can induce neutralizing antibodies and then only with low potency and limited neutralizing breadth. The reasons for vaccine failure may, on one hand, involve the ambiguous conformation of MPER. The native conformation of MPER, or the conformation capable of inducing neutralizing antibodies, has not been determined, and the change of MPER conformation during the membrane fusion process also has not been elucidated. On the other hand, MPER-specific bNAbs possess the cross-reactivity with human autoantigens, and it has been demonstrated that the mechanism of host tolerance mechanism impairs MPER-specific neutralization responses (Kelsoe and Haynes, 2017). These open questions serve to compound the difficulties in designing immunogens and immunization protocols.

This review will focus on recent progress in the field from the following perspectives: (1) MPER structure and its role in membrane fusion, (2) the neutralizing epitopes in MPER and neutralization mechanisms of MPER-specific bNAbs, as well as the limitations in eliciting neutralizing antibodies, and (3) different strategies for MPER vaccine design and current harvests. Understanding the properties and characteristics of MPER structure and immunology, as viewed from these perspectives, will not only be helpful in analyzing how they pose obstacles to vaccine development, but also provide some tentative guidelines for designing reasonable immunogens and vaccines with the hope of ultimately designing an effective HIV vaccine and inducing MPER-specific bNAbs.

## Structure and function of gp41 mper: a dilemma of unknown conformations

**Figure 1:**
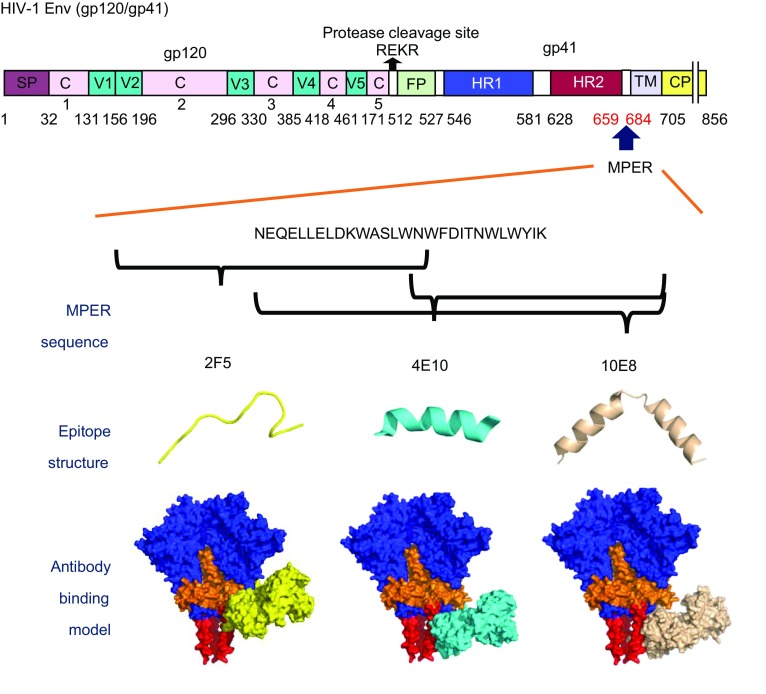
**MPER in the envelope glycoproteins of HIV-1 and conformation of MPER binding to antibodies**. As C-terminus (aa 660–683, HXB2 numbering) of gp41 subunit ectodomain, gp41 MPER bridges the extracellular domain and transmembrane region of Env. The crystal structure of 2F5 Fab in complex with its epitope peptide (PDB ID code: 1TJI) reveals that its epitope forms a β turn conformation, whereas epitope of 4E10 (PDB ID code: 2FX7) forms an α helical conformation. Similar to 4E10, 10E8 forms two α helixes at N- and C-terminus of MPER, respectively. Blue in the figure indicates gp120, orange indicates gp41, and red indicates MPER. The epitope and Fab of 2F5, 4E10 and 10E8 are represented by yellow, cyan and brown, respectively

HIV-1 Env is the sole viral antigen exposed on the virion surface. It is first synthesized as a gp160 glycoprotein precursor and then cleaved into a mature complex constituted by the noncovalent association of three gp120 (surface) and three gp41 (transmembrane) subunits, forming a highly glycosylated trimer of heterodimers (Wyatt et al., 1998; Zanetti et al., 2006; Liu et al., 2008). As C-terminus of the gp41 subunit ectodomain, gp41 MPER bridges the extracellular domain and transmembrane region of Env (Munoz-Barroso et al., 1999; Salzwedel et al., 1999) (Fig. 1), which is a highly conserved motif near the viral envelope surface. The steric hindrance of gp120 and the high hydrophobicity of MPER make MPER partly embedded in the viral membrane (Sun et al., 2008), making it difficult to resolve the native conformation of MPER in the envelope glycoprotein trimer (Lee et al., 2016). In addition, the epitopes of the reported bNAbs reveal quite different conformations so that multiple conformations may be associated with the induction of neutralizing antibodies. During the fusion process, gp41 will undergo dramatic structural changes, which results in continuous contact between the immune system and MPER with different conformations. However, the exact conformations that manifest in the membrane fusion process are still not clear. Thus, the problem of unknown conformations hinders immunogen design.

### Structure of gp41 MPER

The native structure of MPER is still unclear. Previous studies have put forth two different structural models by cryoelectron tomography (Zanetti et al., 2006; Zhu et al., 2006). Zhu et al. have proposed that MPER and the transmembrane (TM) regions, as the stalk of each trimer, are composed of three separate legs that obliquely stretch out of the trimer’s head, much like a tripod. Some researchers hold that such tripod-like model is consistent with the present views concerning gp41 MPER interaction with the membrane (Zhu et al., 2006; Buzon et al., 2010). In contrast, the structural model proposed by Zanetti et al. shows the TM region of simian immunodeficiency virus (SIV) gp41 as a stem in the viral surface. These conflicting structures may be attributed to different methods used to collect the data and/or the computational approaches used to determine the structures (Subramaniam, 2006). The recent study of Dev et al. supports the stem model of TM (Dev et al., 2016). Using cryogenic electron microscopy, Lee et al. analyzed a clade B virus Env lacking only the cytoplasmic tail stabilized by 10E8. The result of nuclear magnetic resonance (NMR) suggests that MPER is embedded in the membrane and that MPER and heptad repeat 2 (HR2) are connected flexibly (Lee et al., 2016). So far, the native conformation of MPER is still fuzzy (Lee et al., 2016) and requires further study.

NMR and surface plasmon resonance (SPR), among other technologies, show that MPER adopts an α helical conformation partially embedded in the viral membrane, consisting of two independent domains separated by a flexible hinge (Sun et al., 2008; Song et al., 2009). These two segments show different membrane-interacting attributes such that the C-terminal domain is embedded in the membrane, and the N-terminal domain is more exposed. Owing to amphiphilic characteristics of the C-terminal domain, the hydrophobic residues are buried in the membrane, whereas the polar residues are solvent-exposed (Huarte et al., 2008; Sun et al., 2008; Song et al., 2009; Kim et al., 2013). Moreover, MPER exhibits different conformations when bound by antibodies (Fig. 1). As shown in Figure 1, the crystal structure of 2F5 Fab in complex with its epitope peptide reveals that the _664_DKW_666_ core motif forms a β turn conformation (Bryson et al., 2001). In contrast, the crystal structure of Fab 4E10 in complex with its epitope peptide was found to form an α helical conformation from D674 to K683 (Cardoso et al., 2005; Cardoso et al., 2007). Similar to 4E10, the crystal structure of Fab 10E8 in complex with its epitope peptide also forms an α helical conformation (Huang et al., 2012). These results indicated that MPER is natively flexible, indicating that more than one structure is associated with neutralization and, at the same time, implying that multiple conformations of MPER immunogens may be favorable to the induction of bNAbs. However, it remains puzzling whether some kind of conformation, or several kinds, can be applied to vaccine design.

### Role of gp41 MPER in the membrane fusion process

The HIV-1 Env transmembrane subunit gp41 serves to anchor the Env protein to cellular membranes and mediate membrane fusion during virus entry into the cell (Chan and Kim, 1998). When the membrane fusion process initiates, gp120 interacts with CD4 molecule on the surface of target cells with a high affinity, which facilitates a series of conformational changes. The gp120 coreceptor binding site is exposed transiently, allowing gp120 attachment to the CCR5 or CXCR4 chemokine receptor (Maddon et al., 1986; McDougal et al., 1986; Rizzuto et al., 1998). Coreceptor ligation triggers the structural rearrangement of gp41, allowing the gp41 fusion peptide (FP) to insert into the target cell membrane, which accounts for a transient prehairpin fusion intermediate, and the cellular and viral membrane are linked by gp41 with an extended conformation. Then α helical domains HR1 and HR2 of each gp41 monomer are reversibly folded into a 6-helix bundle (6-HB) conformation (Su et al., 2017a; Su et al., 2017b), bringing both cellular and viral membrane closer to ultimately generate membrane fusion (Blumenthal et al., 2012; Klasse, 2012).

The entire gp41 is mostly occluded by gp120 in the native virus spike where MPER is exposed transiently in the fusion process (Dimitrov et al., 2007). As such, the importance of MPER in the function of Env is highlighted by analyses of mutant viruses involving MPER deletions, insertions and substitutions (Munoz-Barroso et al., 1999; Dimitrov et al., 2007; Vishwanathan and Hunter, 2008). For example, substitution of the five conserved tryptophan residues in MPER greatly compromises the integration of gp41 into virions and, consequently, blocks viral entry (Munoz-Barroso et al., 1999). In addition, deletion of the 666WASLWNWFDITNWLWYI682 region completely abolishes the formation of syncytium. Such evidence shows that MPER plays an important role in HIV-1 Env-mediated fusion and virus infection, which is consistent with the high conservatism of its sequence (Salzwedel et al., 1999). In addition, some studies has indicated that MPER may mediate membrane partition, fusion and penetration (Suarez et al., 2000a; Suarez et al., 2000b). MPER plays a key role in membrane destabilization by interacting with the lipid membrane (Bellamy-McIntyre et al., 2007). The high content of tryptophan may enable MPER to interact with the lipid membrane and destabilize it (Suarez et al., 2000b; Stano et al., 2005). Some studies have also revealed that MPER plays a role in HIV-1 CD4-independent viral transcytosis at the epithelial barrier (Bomsel, 1997) where the conserved sequence 662ELDKWA667 interacts with galactosyl ceramide receptors (Alfsen and Bomsel, 2002), indicating that MPER is functional in the mucosal infection of viruses. The secretory IgA from cervicovaginal secretions of HIV-1-infected individuals can block viral transcytosis though binding the 662ELDKWA667 sequence (Alfsen et al., 2001; Leroux-Roels et al., 2013), indicating that the use of an immunogen containing MPER is likely to induce vaginal IgA with transcytosis-blocking activity, a finding also confirmed by another report (Bomsel et al., 2011). Therefore, eliciting antibodies against MPER by vaccination may disturb its function thus effectively block viral entry and protect humans from HIV-1 infection.

To sum up, a number of groups have shown that MPER is conserved and plays important roles in the course of viral infection. However, researchers have not yet determined the exact structure of gp41, the native conformation of MPER, or the conformation of MPER capable of inducing neutralizing antibodies, let alone the allosteric mode of gp41, especially MPER during the membrane fusion process. Consequently, we have a dilemma of unknown conformations that seriously militates against successful immunogen design. Apart from such parameters as low accessibility and unknown conformations, the host tolerance mechanism also influences MPER-specific neutralization responses. We will discuss the humoral responses targeting MPER in detail below.

## Humoral responses targeting mper: coexistence of hopes and limitations

In the earlier clinical trials, HIV-1 Env-based subunit vaccines were tested to elicit antibodies specific for gp120. However, these antibodies had no neutralizing activity and vaccinated people were not protected from HIV infection. The failure of these trials promoted a shift to the development of HIV vaccines for eliciting T cell responses. However, the disappointing outcome from the clinical trials of a T cell-based vaccine regimen, the STEP trial, conducted by Merck and HIV Vaccine Trials Network (HVTN), has dealt another setback to AIDS vaccine development (Miedema, 2008). The failure of the STEP trial further reinforced the notion that an effective AIDS vaccine needs to induce both strong CTLs (cytotoxic T lymphocytes) and bNAbs against HIV infection (Barouch, 2008; Fauci et al., 2008; Walker and Burton, 2008). Moreover, as mentioned in the section above, the contribution of IgAs at the mucosal surface also should not be ignored (Bomsel, 1997).

Nevertheless, efforts to engineer vaccines that can induce HIV bNAbs have encountered great difficulties; no one can induce bNAbs by immunization with immunogens containing MPER sequence(s). To gain a better understanding of this, we will analyze (1) the neutralizing epitopes in MPER and neutralization mechanisms of MPER-specific bNAbs and (2) limitations in the elicitation of neutralizing antibodies. The neutralization mechanisms of bNAbs highlight the importance of membrane and show the role of lipids as a native scaffold to shape the structure of MPER, in turn suggesting the importance of lipids in immunogen design. In addition, the limitations of inducing neutralizing antibodies put more burdens on vaccine design.

### Epitopes and neutralization mechanisms of three bNAbs

The mAbs isolated from HIV-1-infected individuals are the strongest evidence proving that the human immune system can generate MPER-specific neutralization responses. Multiple mAbs targeting MPER have been isolated so far, such as 2F5, 4E10, Z13, Z13e1, m66.6, CH12 and 10E8 (Muster et al., 1993; Muster et al., 1994; Stiegler et al., 2001; Zwick et al., 2001; Nelson et al., 2007; Hessell et al., 2010; Morris et al., 2011; Huang et al., 2012; Ofek et al., 2014). Among these antibodies, 2F5, 4E10 and 10E8 reveal broadly neutralizing activity, and as such, they have been explored more thoroughly (Table 1).

**Table 1:** Features of the reported bNAbs against MPER.

Antibody	Binding sequence	No. of viruses	IC_50_ < 50 μg/mL	IC_50_ < 1 μg/mL	Mean IC_50_ (μg/mL)
2F5	_656_NEQELLELDKWASLWN_671_	177	57%	16%	1.92
4E10	_671_NWFDITNWLWYIK_683_	181	98%	37%	1.3
10E8	_664_DKWASLWNWFDITNWLWYIK_683_	180	98%	72%	0.22

2F5 and 4E10 are among the first bNAbs discovered that were generated by electrofusion of peripheral blood mononuclear cell mixtures from different HIV-1-infected individuals (Buchacher et al., 1994). 2F5 targets the sequence_656_NEQELLELDKWASLWN_671_ within the N-terminus of MPER (Muster et al., 1993), of which the central core,_664_DKW_666_, is crucial to neutralization, as demonstrated by alanine-scanning mutagenesis assays (Zwick et al., 2005). The crystal structures of 2F5 in complex with a synthesized short or long peptide based on its epitope have been analyzed, and the results showed that the _664_DKW_666_ core motif presents a β turn conformation. The structure of 2F5 in complex with the long peptide reveals that only 41% of its sequence binds 2F5 with some unbound hydrophobic regions, which may be subject to the steric hindrance of Env or embedded in the lipid membrane (Ofek et al., 2004; Bryson et al., 2008). 2F5 has a relatively high potency and can neutralize 57%–67% of viral isolates with a concentration causing 50% inhibition of the desired activity (IC_50_) below 50 µg/mL (Binley et al., 2004; Huang et al., 2012). However, as a result of a mutation in the central core epitope (DSW instead of DKW), HIV-1 C subtype viruses are usually 2F5-resistant (Bures et al., 2002; Binley et al., 2004; Gray et al., 2006).

4E10 targets the distal conserved tryptophan-rich motif that is located C-terminal to the 2F5 epitope, including the sequence_671_NWFDIT_676_, and extending toward C-terminal residues where W672, F673, I675, T676, L679 and W680 have the most important contact with the antibody (Zwick et al., 2001). Although presenting a moderate potency, 4E10 displays a remarkable breadth to neutralize 98%–100% viral isolates with an IC_50_ below 50 µg/mL (Binley et al., 2004; Walker et al., 2009). Compared with the pseudoviruses obtained in 293 T cells, further characterization of 2F5 and 4E10 revealed their reduced potency against transmitted/founder viruses (T/F IMC) or replicating viruses obtained from primary lymphocytes (Louder et al., 2005; Provine et al., 2009; Provine et al., 2012; Miglietta et al., 2014). In spite of these possible limitations, both 2F5 and 4E10 were shown to protect nonhuman primates (NHP) against viral challenge (Mascola et al., 2000; Hessell et al., 2010), and no major clinical complication arose when administered to human recipients (Trkola et al., 2005).

In order to delineate a complete map of HIV-1 neutralizing determinants, substantial efforts have been made to isolate new bNAbs since 2009. The development of high-throughput analysis of single memory B cells and the use of fluorescently labeled Env-based protein probes to isolate antigen-specific B cells have significantly contributed toward the discovery of new HIV-1 neutralizing antibodies (Doria-Rose et al., 2009; Scheid et al., 2009; Wu et al., 2010). In this context, mAb 10E8 discovered in 2012 proved once again that important bNAbs targeting this area can be generated. It also prompted researchers to consider MPER as a major vaccine target (Huang et al., 2012).

10E8 targets the sequence_656_NEQELLELDKWASLWN_671_ within the C-terminus of MPER, which overlaps the epitopes of 2F5 and 4E10. It neutralized 98% of 181 pseudoviruses with an IC_50_ below 50 µg/mL, showing a mean IC_50_ of 0.25 µg/mL for the sensitive viruses, while mean IC_50_ values of 4E10 and 2F5 were 1.3 and 1.92 µg/mL, respectively. Interestingly, 72% of the panel was neutralized by 10E8 with an IC_50_ below 1 µg/mL, while the percentages of 4E10 and 2F5 were 37% and 16%, respectively (Huang et al., 2012). Therefore, 10E8 can neutralize viruses with a greater potency and breadth than the previously discovered 2F5 and 4E10, and it is comparable to some of the most potent bNAbs, such as VRC01 or PG9/PG16 (West et al., 2014). Notably, 10E8 was also reported to protect NHP against viral challenge (Pegu et al., 2014).

Just recently, a new lineage of MPER-specific bNAbs, designated DH511, was isolated from memory B cells and plasma of an HIV-1-infected donor (Williams et al., 2017). The DH511 lineage, which is derived from the same heavy chain germline gene family (VH 3–15) as 10E8, presents long CDR H3 loops of 23 to 24 amino acids, and the somatic mutation rates of VH and VL are 15%–22% and 14%–18%, respectively. DH511.2, as the most potent mAb of this clone lineage, neutralized 206 out of 208 pseudoviruses of a geographically and genetically diverse panel with a median IC_50_ of 1 µg/mL, being slightly broader, but less potent, than 10E8 (Williams et al., 2017).

Independent of their origin, all these antibodies are the product of a long process of affinity maturation, which is highly mutated with an unusually long and hydrophobic heavy chain complementary determining region 3 (CDR H3) (Zwick et al., 2004; Cardoso et al., 2005; Huang et al., 2012). In addition, these antibodies share a similar neutralization mechanism. Although some residues of the CDRs are very important for binding peptidic epitopes, researches have shown that the most hydrophobic loops directly interact with membrane lipid (Alam et al., 2007; Alam et al., 2009; Lutje Hulsik et al., 2013). In previous study, 2F5 was predicted to bind lipids via CDRL1 and CDRH3 (Julien et al., 2008). Recently, lipid-binding sites of 4E10 and 10E8 were determined by X-ray crystallography (Irimia et al., 2016; Irimia et al., 2017). The bNAbs against MPER binding to a peptide sequence obey the Langmuir curve model, but SPR-based studies demonstrated that binding polypeptide-membrane complex follows a two-step (encounter–docking) model. First, the antibody attaches to the lipid membrane through its long hydrophobic CDR H3 and concentrates around the MPER epitope. Once conformational changes take place, the antibody binds to the pre-hairpin intermediate of gp41 (Alam et al., 2007; Alam et al., 2009). The mechanism facilitates the approach of antibody to epitope, overcoming the poor exposure of MPER and also taking advantage of its close proximity to the viral membrane. However, the exact neutralization mechanism is still controversial and requires further exploration (Frey et al., 2008; Kim et al., 2014).

In sum, all epitopes of bNAbs targeting MPER may be composed of peptidic residues and membrane lipids together. The importance of membrane in the neutralization mechanism of bNAbs shows the important role of lipids as a native scaffold in shaping the structure of MPER, thus indicating the significance of lipids in immunogen design. Therefore, in order to generate MPER-specific neutralization responses, the membrane environment may be required to present the neutralizing determinant properly.

### Generation of neutralizing antibodies: limited by polyreactivity/autoreactivity

Up to now, the reported bNAbs against MPER have a prevalence of polyreactivity and autoreactivity. In 2005, the polyspecific binding of 4E10 and 2F5 mAbs to cardiolipin and other anionic phospholipids was reported (Haynes et al., 2005a). Furthermore, conserved host antigens bound by 2F5, 4E10 and 10E8 were also identified (Yang et al., 2013; Liu et al., 2015). 2F5 binds to the enzyme kynureninase (KYNU) containing the same sequence (ELDKWA) as its epitope, which is highly conserved in different mammal species. 4E10 binds to splicing factor-3b subunit-3 and type I inositol triphosphate (IP3R1) (Yang et al., 2013). Although considered as non-polyreactive initially, subsequent studies indicated that 10E8 possibly needs to bind membrane lipids, especially cholesterol, to mediate neutralization (Huang et al., 2012; Chen et al., 2014; Irimia et al., 2017). Recently, the crystal structure of 10E8 in complex with MPER shaped by a scaffold revealed that its complete epitope consists of MPER and lipids (Irimia et al., 2017). Although described as non-autoreactive initially, 10E8 also recognizes FAM84A protein (Liu et al., 2015); however, such recognition did not seem to cause strong toxicity *in vivo* since clinical trials showed 2F5, 4E10 and 10E8 to be relatively safe (Trkola et al., 2005; Pegu et al., 2014).

These studies reporting on polyreactivity and autoreactivity suggest that autoreactive B cells that cross-react with MPER sequences may be impaired in the native repertoire. Thus, this immunologic tolerance mechanism might be associated with HIV-1 evasion of immune responses (Haynes et al., 2005b; Verkoczy et al., 2014). This hypothesis was confirmed by monitoring B cell development in knockin (KI) mouse models carrying V (D) J rearrangements identical to those of the mature bNAbs 2F5 and 4E10. These models showed a normal early B cell development, but a blockade from pre-B to immature IgM^+^ B cells at the first tolerance checkpoint (Verkoczy et al., 2010; Doyle-Cooper et al., 2013; Verkoczy et al., 2013; Verkoczy and Diaz, 2014). B cell central tolerance takes place in the bone marrow, hindering the development of autoreactive B cells by several mechanisms, such as clonal deletion and receptor editing (Nemazee, 2017). After that, some autoreactive B cells can still migrate from the bone marrow as anergic cells, showing a hyporesponsive state and a shortened lifespan. However, under special circumstances, the anergic B cells can be activated and differentiate into antibody-producing B cells (von Boehmer and Melchers, 2010). Consistent with this phenomenon, when 2F5 KI mice were immunized with MPER peptide-liposome immunogens, anergic B cells could be restored to generate specific neutralizing antibodies (Dennison et al., 2009; Verkoczy et al., 2011). More recently, a 2F5 germline knock-in (KI) mice model has demonstrated that remaining anergic B cells can also be activated by germline-mimicking immunogens when 2F5 precursors are deleted (Zhang et al., 2016). All these results indicated that the production of 2F5 and 4E10 antibodies may be controlled by immunologic tolerance mechanisms (Yang et al., 2013; Liu et al., 2015).

Impairment of autoreactive B cells that cross-react with MPER sequences in the native repertoire can also explain the low frequency of MPER neutralizing antibodies during the course of natural infection (Haynes et al., 2005a; Haynes et al., 2005b; Kelsoe and Haynes, 2017). The characterization of different cohorts in Europe, America and South Africa indicated that MPER-specific neutralizing responses are less represented compared with other epitopes during natural infection. For example, in a South African cohort of 156 HIV-1-infected individuals, only three showed high titers of anti-MPER antibodies (Gray et al., 2009), and depletion of these antibodies resulted in the loss of neutralization breadth. A recent study analyzed the neutralization profile of 439 plasma samples and demonstrated far less prevalence of MPER-specific antibodies compared with other epitopes, mainly the V3 N332-dependent glycan supersite (Landais et al., 2016).

**Figure 2:**
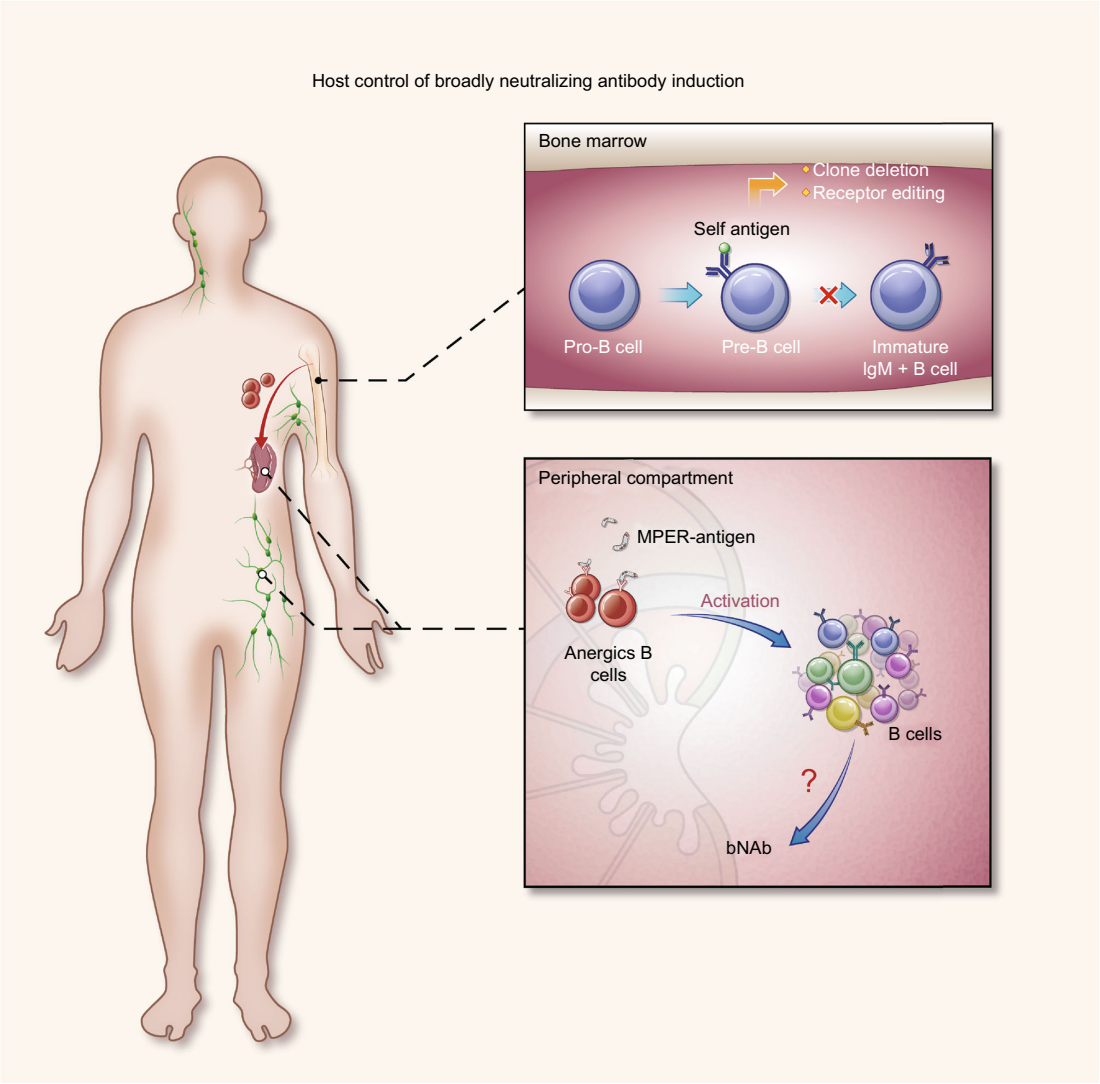
**Host control of bNAbs induction**. When developing in the bone marrow, pre-B cells that possibly produce bNAbs later always bind the lipids (or other autoantigens); therefore most of them are removed by clonal deletion and receptor editing and accordingly cannot develop into immature IgM^+^ B cells. However, a few lipid-reactive (or other autoantigens) B cells can still migrate from the bone marrow to the secondary lymphoid organ as anergic cells which can be activated again by antigens, such as MPER-lipid complex, similar to lipids (or other autoantigens), and differentiate into antibody-producing B cells. Since only a small number of anergic cells can move to the secondary lymphoid organ, the difficulty of generating bNAbs cannot be understated

Judging from the results of these studies, we might assume the following steps (Fig. 2). When developing in the bone marrow, pre-B cells that possibly produce bNAbs later always bind lipids (or other autoantigens); therefore most of them are removed by clonal deletion and receptor editing and accordingly cannot develop into immature IgM^+^ B cells. However, a few lipid-reactive (or other autoantigens) B cells can still migrate from the bone marrow to the secondary lymphoid organ as anergic cells which can be activated again by antigens, such as MPER-lipid complex, similar to lipids (or other autoantigens), and differentiate into antibody-producing B cells. Since only a small number of anergic cells can move to the secondary lymphoid organ, the difficulties of generating bNAbs are far-reaching. Therefore, how anti-MPER bNAbs can be induced is still a key issue worthy of consideration.

## Vaccine design targeting mper: walk towards the sun

**Figure 3:**
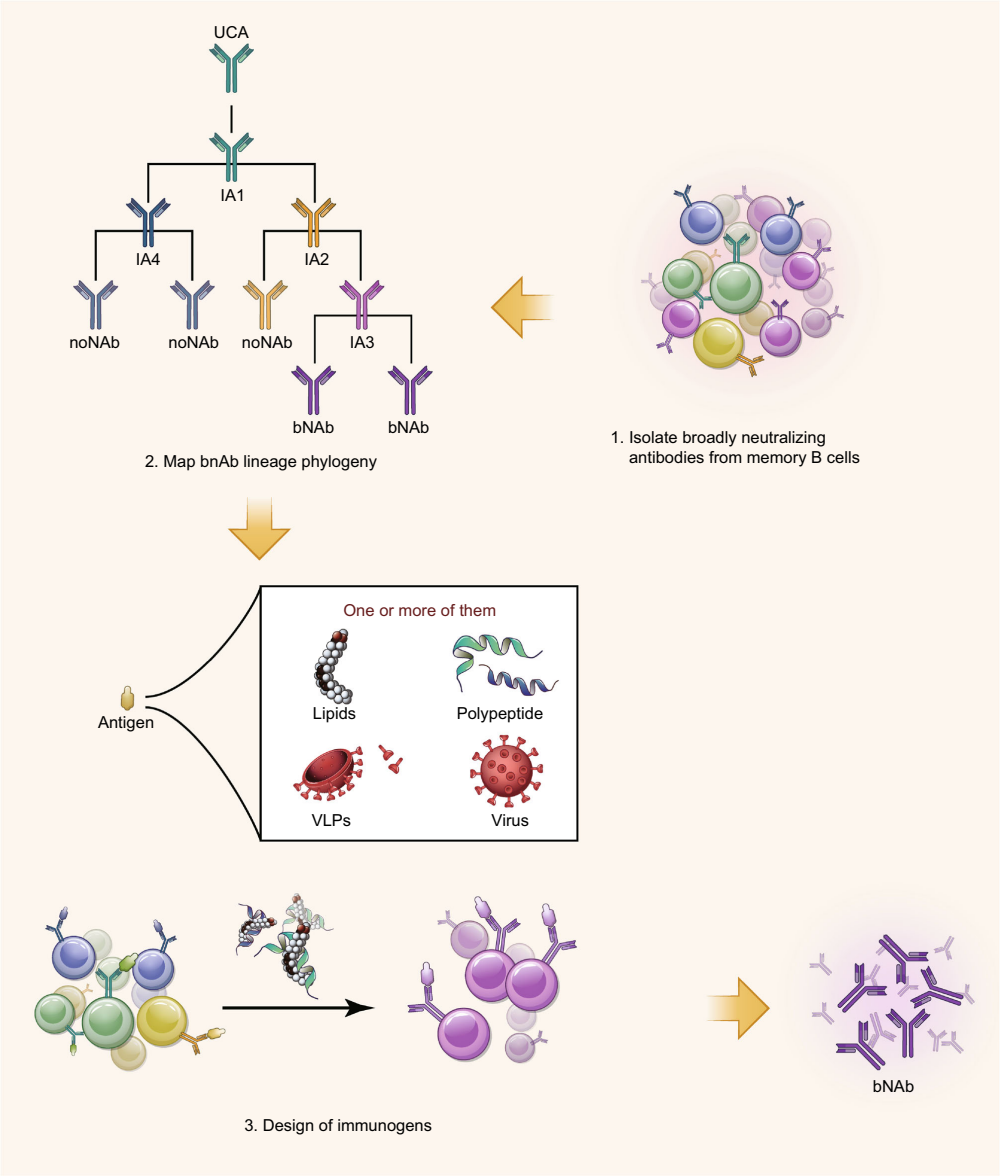
**Schematic diagram representing a possible strategy to induce bNAbs against MPER**. First, affinity-matured, bNAbs and their precursors against MPER are isolated from HIV-1-infected donors, using methods such as memory B cell cultures or antigen-specific B cell sorting. Second, based on known bNAb sequences, next-generation sequencing can be used to retrieve numerous V_H_DJ_H_ and V_L_J_L_ clonally related rearrangements. If appropriate longitudinal samples are available, it is possible to define the full lineage phylogeny and infer the unmutated common ancestor (UCA) and early maturation intermediate antibodies (IAs). Third, recombinant monoclonal antibodies expressing the bNAb precursor V_H_DJ_H_ and V_L_J_L_ rearrangements from UCA to IAs can be used to design MPER-based immunogens. Different kinds of immunogens should be included, such as MPER-based peptide, lipids and VLPs or pseudoviruses. MPER-based peptide should be properly combined with lipids, thus potentially presenting a conformation capable of engaging B cells and inducing neutralizing antibodies

Despite many efforts, bNAbs targeting MPER still cannot be induced by immunization. Only a few vaccine candidates were found to induce neutralizing antibodies, albeit with low potency and limited breadth. Initially, bNAb-binding amino acid sequences were introduced into fusion proteins, peptide-based proteins or chimeric viruses, attempting to induce 2F5 or 4E10-like antibodies (Montero et al., 2008), but only MPER-specific antibodies with no neutralizing activity were produced. Therefore, beyond the recognition of specific peptidic sequences within MPER, additional variables should be considered. The common characteristics revealed by anti-MPER bNAbs, such as lipid reactivity (Frey et al., 2008; Alam et al., 2009), indicate that similar antibodies could be obtained by presenting MPER-based immunogens with a proper conformation in a membrane-like environment. Therefore, as shown in Fig. 3, when it comes to MPER-based vaccine design, at least two aspects should be considered: (1) which conformation would most likely induce anti-MPER bNAbs and (2) what role membrane lipids play in shaping the structure of MPER. The latter aspect suggests that the corresponding immunogen design should take into account accurate lipid components and adjuvant systems (Molinos-Albert et al., 2017b).

Recently, a B-cell lineage-based approach for vaccine design was developed progressively, but not yet applied to MPER (Soto et al., 2016; Bonsignori et al., 2017; Williams et al., 2017). Figure 3 is a schematic diagram representing a possible strategy to induce bNAbs against MPER on the basis of a comprehensive consideration of these two aspects. First, affinity-matured bNAbs and their precursors against MPER would be isolated from HIV-1-infected donors, using methods such as memory B cell cultures or antigen-specific B cell sorting. Second, based on known bNAb sequences, next-generation sequencing could be used to retrieve numerous V_H_DJ_H_ and V_L_J_L_ clonally related rearrangements. If appropriate longitudinal samples are available, it would be possible to define the full lineage phylogeny and infer the unmutated common ancestor (UCA) and early maturation intermediate antibodies (IAs). Third, recombinant monoclonal antibodies expressing the bNAb precursor V_H_DJ_H_ and V_L_J_L_ rearrangements from UCA to IAs could then be used to design MPER-based immunogens. In light of studies on influenza vaccine development, it has been shown that bNAbs against the stem region of the HA could be induced by vaccinating animals with HA from different antigenic lineages (Ye et al., 2011). This approach may also be applied to overcome the weakness of low immunogenicity of MPER-based HIV vaccine. For example, cross prime-boost immunizations with MPER antigens from different HIV-1 subtypes may induce enhanced bNAb responses. Zolla-Pazner et al. have demonstrated that cross prime-boost immunizations with antigens containing six V1V2 sequences and nine scaffold proteins from different HIV-1 subtypes (B, C, E) have induced bNAb responses against infection of HIV, SIV and SHIV (Zolla-Pazner et al., 2016). Furthermore, different kinds of immunogens, such as MPER-based peptide, lipids and VLPs or pseudoviruses may also be tested. MPER-based peptide should be properly combined with lipids to present an appropriate conformation capable of engaging B cells and inducing neutralizing antibodies.

To address the problem of proper MPER conformation to induce bNAbs and the role of membrane lipids for immunogen design, corresponding explorations have already been carried out, as shown in Table 2. In recent years, other viral proteins, as a scaffold, or modified HIV-1 Env were used to explore the appropriate conformation of MPER capable of inducing neutralizing antibodies. For instance, Phogat et al. (2008) utilized S1 protein of hepatitis B virus (HBV) fused with MPER to immunize mice and rabbits. Although anti-MPER antibodies were generated, antiserum did not present neutralizing activity. P15 of porcine endogenous retrovirus presents a structure similar to that of HIV-1 gp41. Accordingly, Strasz et al. (2014) replaced E1 and E2 of P15 with FPPR and MPER of HIV-1, respectively, and 2F5-like antibodies were elicited, albeit without neutralizing activity, after immunizing rats, guinea pigs and goats. However, Luo et al. (2006) also utilized P15 of porcine endogenous retrovirus to replace E2 region with MPER, and the antiserum could neutralize HIV pseudoviruses at 1:20. When it comes to modified HIV-1 Env, such as replacing the loop between NHR and CHR with 2F5 epitope directly (Vassell et al., 2015), replacing the loop with GGGGS sequence (Habte et al., 2015), or deleting the cleavage site of gp120 and gp41 and fusion peptide (Dennison et al., 2011), antibodies without neutralizing activity were all detected in different kinds of antisera immunized with these modified proteins. Banerjee et al. (2016) constructed a gp41-HR1-54Q immunogen which was expected to induce neutralizing antibodies by reducing the stability of 6-HB to simulate gp41 fusion intermediate state. The serum from immunized rabbits also had MPER-specific antibodies, but without neutralizing activity.

**Table 2:** HIV-1 gp41 MPER-targeting vaccines under development. NR, not reported in the literature.

No.	Mimic epitope	Vector	Animal model	Vaccination regimen	Specific antibody	HIV-1 neutralization	References
1	MPER	Porcine endogenous retrovirus (PERV) p15 fragment	Rabbit	Heterogeneous prime-boost (chimeric virus + protein)	MPER-specific antibodies	Partial neutralizing activities for pseudoviruses	Luo et al., 2006
2	MPER	Adenovirus (Ad)	Mouse	Homogeneous prime-boost (chimeric virus)	MPER-specific antibodies	NR	Matthews et al., 2010
3	2F5 or 4E10 epitope/MPER region	Vesicular stomatitis virus (VSV)	Rabbit	Intramuscular injection Heterogeneous prime-boost (chimeric virus + DNA)	MPER-specific antibodies	No	Lorenz et al., 2014
4	MPER	*Lactobacillus acidophilus*	Mouse	Intragastric immunization Homogeneous prime-boost (chimeric virus)	MPER-specific antibodies in serum and mucosal secretions	NR	Kajikawa et al., 2015
5	2F5 or 4E10 epitope/MPER domain	Bovine papillomavirus (BPV) virus-like particles (VLPs)	Mouse	Oral administration Homogeneous prime-boost (chimeric virus)	Epitope-specific serum IgGs and mucosal secretory IgAs	Partial neutralizing activities for HIV-1 (clade B/C)	Zhai et al., 2013
6	2F5 epitope/full-length MPER	Reovirus	Mouse Rabbit	Homogeneous prime-boost (chimeric virus)	No MPER-specific antibodies	No	Boehme et al., 2016
7	2F5/4E10 epitope	Chimeric HIV-gag virus-like particles (VLPs)	Mouse	Intramuscular injection/intranasal immunization Heterogeneous prime-boost (VLPs + DNA)	High levels of mucosal MPER-specific antibodies	NR	Jain et al., 2010
8	2F5/4E10 epitope	Influenza A virus	Guinea pig	Intranasal immunization Homogeneous prime-boost (chimeric virus)	MPER-specific antibodies	Weak and partial neutralizing activities for HIV-1 (clade B/BC)	Zang et al., 2015
9	4E10 epitope	Human rhinovirus (HRV)	Human ICAM-1 transgenic (hICAM-1 Tg) mouse	Intranasal immunization Homogeneous prime-boost (chimeric HRV)	MPER-specific antibodies	Partial neutralizing activities for HIV-1 (clade B/C)	Yi et al., 2015
10	2F5/4E10 epitope	Human rhinovirus (HRV)	Guinea pig	Subcutaneous injection Heterogeneous prime-boost (chimeric virus + peptide)	MPER-specific antibodies	Neutralizing activities for HIV-1 pseudoviruses	Yi et al., 2013
11	2F5 epitope	Adenovirus type 5 (Ad5)	Mouse	Intramuscular injection Homogeneous prime-boost	MPER-specific antibodies	Neutralizing activities for HIV-1 strains	Ura et al., 2009
12	2F5 epitope	Coxsackievirus B4 (CVB4)	Mouse	Intraperitoneal (IP) injection/oral immunization Homogeneous prime-boost (chimeric virus)	Anti-gp41 antibodies	NR	Gu et al., 2012
13	10E8 epitope	Live attenuated *Salmonella*	Mouse	Oral administration Heterogeneous prime-boost (bacterium + peptide)	MPER-specific antibodies in serum and mucosa	Neutralizing activities for HIV-1 SF162 pseudoviruses	Li et al., 2016
14	gp41	Influenza HA protein	Guinea pig	Intramuscular injection Homogeneous prime-boost (DNA/VLPs)	Anti-gp41 antibodies	Partial neutralizing activities for HIV	Ye et al., 2011
15	MPER	Hepatitis B surface antigen (HBsAg)	Mouse	Homogeneous prime-boost (HBsAg-MPER particles)	MPER-specific antibodies	No	Phogat et al., 2008
			Rabbit	Heterogeneous prime-boost (HBsAg-MPER) + (Env-PLs)	Anti-Env antibodies	Neutralizing activities for HIV-1 isolates	
16	MPER	Foamy virus Bet protein	Rat	Homogeneous prime-boost (hybrid protein)	MPER-specific antibodies	No	Muhle et al., 2013
17	2F5/4E10 epitope	Porcine endogenous retrovirus (PERV) p15E protein	Rat Guinea pig Goat	Homogeneous prime-boost (hybrid protein)	MPER-specific antibodies	No or very weak neutralizing activities	Strasz et al., 2014
18	MPER	*Geobacillus stearothermophilus* E2 protein	Rabbit	Homogeneous prime-boost (E2 particles + gp160 DNA)	MPER-specific antibodies	Low and partial neutralizing activities for HIV	Krebs et al., 2014
19	4E10/10E8 epitope	Norovirus P particles (NoV PPs)	Mouse Guinea pig	Homogeneous prime-boost (protein)	MPER-specific antibodies	Neutralizing activities for HIV pseudoviruses	Yu et al., 2015
20	MPER	gp140	Guinea pig Rhesus macque	Heterogeneous prime-boost (gp140 oligomer + MPER peptide-liposomes)	MPER-specific antibodies	No	Dennison et al., 2011
21	gp41 six-helix bundle (6-HB)/MPER	N- and C-terminal heptad repeats and the MPER domain (NCM)	Rabbit	Subcutaneous injection Homogeneous prime-boost (protein)	MPER-specific antibodies	Partial neutralizing activities	Wang et al., 2011
22	gp41	TPA leader sequence Influenza strain H3 HA2 protein *Saccharomyces cerevisiae* GCN4 protein	Rabbit	Intramuscular injection Heterogeneous prime-boost (DNA + VLP proteins)	Weak MPER-specific antibodies	Weak and low neutralizing activities for viral isolates (clade B/C)	Benen et al., 2014
23	gp41 6-HB	gp41-HR1-54Q	Rabbit	Homogeneous prime-boost (protein)	MPER-specific antibodies	No	Habte et al., 2015
24	gp41 fusion intermediates	gp41 N- and C-heptad repeats + MPER	Rabbit	Heterogeneous prime-boost	MPER-specific antibodies	No	Vassell et al., 2015
25	MPER	gp120 V1/2 region	Mouse Rabbit	Heterogeneous prime-boost (DNA + protein)	High levels of gp120-specific antibodies No MPER-specific antibodies	Neutralizing activities for homologous neutralization-resistant JR-FL virus	Law et al., 2007
26	MPER + lipid	Liposomes + phosphatidylinositol-4-phosphate (PIP)	Mouse	Intraperitoneal injection Homogeneous prime-boost	MPER-specific antibodies	Partial neutralizing activities for HIV-1	Matyas et al., 2009
27	MPER	Liposomes containing chemically modified peptides	Rabbit	Homogeneous prime-boost (liposomes)	MPER-specific antibodies	No	Venditto et al., 2014
28	gp41 fusion intermediates	Destabilized 6-HB	Rabbit	Homogeneous prime-boost (protein)	MPER-specific antibodies	No	Banerjee et al., 2016
29	MPER-V3	MPG	Mouse	Homogeneous/ heterogeneous prime-boost (DNA + peptide)	MPER-specific antibodies	NR	Bolhassani et al., 2015
30	EC26-2A4 epitope within MPER	Sequential oligopeptide carriers (SOC)/palmitoyl acid	Mouse	Intramuscular injection Heterogeneous prime-boost	MPER-specific antibodies	Neutralizing activities for HIV-1 SF162	Zhou et al., 2012
31	10E8 epitope	Immunogenic peptide T10HE and T10E	Mouse	Heterogeneous prime-boost (peptide + pseudoviruses)	10E8-like neutralizing antibodies	Neutralizing activities for homogeneous HIV-1	Yu et al., 2014
32	2F5 epitope	Anti-idiotypic antibody Ab2/3H6 fused with immune-modulators	Rabbit	Homogeneous prime-boost (protein)	MPER-specific antibodies	No	Mader and Kunert, 2012
33	2F5 epitope	Three tandem 2F5 epitope repeats	Mouse	Intranasal injection Homogeneous prime-boost (liposomes)	MPER-specific antibodies	Neutralizing activities for HIV-1 primary isolates	Mohan et al., 2014
34	10E8 epitope	Four tandem 10E8 epitope repeats	Rabbit	Intradermal immunization Homogeneous prime-boost (peptide)	MPER-specific antibodies	Neutralizing activities for pseudoviruses	Sun et al., 2016

In general, the neutralizing activity of antibodies obtained by immunizing with protein scaffolds alone was unsatisfactory, possibly because other characteristics, such as membrane binding, were not addressed in the design of these scaffolds. Therefore, more and more research interest has focused on lipid-containing immunogens. Experimental data showed that membrane lipids may modulate the structure of MPER by promoting a native-like conformation, and such membrane lipids were demonstrated to improve immunogenicity (Hanson et al., 2015; Molinos-Albert et al., 2017a). In particular, it was proved that lipids overexpressed in the viral membrane, such as cholesterol and sphingomyelin, may induce higher antibody titers, compared with common POPC lipids (Molinos-Albert et al., 2017a).

In the lipid-containing immunogens, peptide-based vaccine regimens occupy a certain proportion. Matyas et al. (2009) utilized liposomes containing a synthetic MPER peptide as a peptide antigen, phosphatidylinositol-4-phosphate (PIP) as a lipid antigen, and monophosphoryl lipid A as a potent adjuvant to immunize mice. Anti-MPER and anti-PIP antibodies were generated from which IgM mAb was isolated that not only could recognize 2F5 and 4E10 epitopes and bind to PIP, but also could present a certain neutralizing capacity for HIV-1 virions in human peripheral blood. Venditto et al. (2014) synthesized the full-length MPER peptide and modified the single amino acid site of MPER chemically (phosphorylation, sulfation or nitrification). The modified peptide was presented in liposome to immunize rabbits. Higher titer antibodies were induced, but without neutralizing activity. Mohan et al. (2014) designed a liposome immunogen containing three tandem 2F5 epitope repeats and defensins to immunize mice intranasally. Antiserum and mucosal system of mice all generated high titer IgG and IgM antibodies, and antiserum showed high neutralizing activity for an original isolate. Donius et al. used antigen-coupled liposome to immunize mice, and MPER-specific antibodies were isolated from the long-lived bone marrow plasma cells. These antibodies were produced under the selective pressure of MPER in the context of lipids, but they did not reveal any characteristic of polyreactivity (Donius et al., 2016).

Except for the peptide-based vaccine, chimeric viruses or virus-like particles (VLPs) may be better platforms as a result of taking the conformation of MPER peptide and the scaffold feature of lipids into consideration. With the research progresses in recent years, more viral vectors have been designed to express HIV-1 neutralizing epitope, such as adenovirus (Ura et al., 2009), influenza (Ye et al., 2011; Zang et al., 2015) and rhinovirus (Yi et al., 2013). These viral vectors can express and expose the chimeric peptidic epitopes and induce antibodies to some extent, but only a few of them could induce HIV-1 neutralizing antibodies. Ura et al. (2009) adopted adenovirus Ad5 as the vector and inserted 2F5 epitope sequence in the HVRS region of envelope protein. The antiserum of mice immunized with chimeric viruses could neutralize various strains of HIV-1. Moreover, 2F5-like antibodies were generated, and mAb targeting MPER was purified and verified. This mAb did, indeed, have the capacity to neutralize HIV-1. In view of the strong immunogenicity of influenza, Ye et al. (2011) fused HIV-1 gp41 at the C-terminus of influenza HA1 subunit and immunized new guinea pigs by HA/gp41 plasmid or VLPs. Anti-MPER antibodies were elicited, and antiserum could neutralize HIV pseudoviruses expressing SIV Env with chimeric 4E10 epitope. Such neutralizing capacity could also be blocked by the MPER peptide, indicating that the immunogen based on HA/gp41 produced anti-MPER antibodies with some neutralizing activity. Similarly, Zang et al. (2015) inserted 2F5 and 4E10 epitopes into the linker domain between the trimeric core structure and the transmembrane domain of influenza A virus HA2 and immunized guinea pigs with chimeric viruses. The serum exhibited a weak neutralizing activity for HIV-1 clade B and clade BC. Yi et al. (2013) utilized the rhinovirus as the vector to present the 2F5 and 4E10 peptidic epitopes and immunized mice with human rhinovirus receptor hICAM-1. The antiserum could recognize and neutralize HIV-1. Meanwhile, it was shown that the existing anti-rhinovirus antibodies could be avoided by nasal immunization without influencing the presentation of antigen epitope on the rhinovirus vector.

To some extent, the titer and neutralizing activity of antibodies induced by chimeric viruses and VLPs are indeed superior to the protein vaccine and peptide-based vaccine, but bNAbs are still not elicited, as expected, to protect humans powerfully from HIV-1 infection. The complicated features of MPER, such as structure and immunology, still constitute the main stumbling blocks against the development of a successful vaccine.

## Conclusions and prospects

In conclusion, recent substantial progresses involving the analyses of structure and immunology of MPER, particularly, the structure of this region bound by three MPER-specific bNAbs (2F5, 4E10 and 10E8), their epitopes, and their neutralization mechanisms. However, no one can induce bNAbs targeting MPER by immunization, which, according to the most recent studies, can largely be attributed to two key problems. On the one hand, unknown conformations confound vaccine design against MPER. Neither native MPER conformation nor the conformation capable of inducing neutralizing antibodies has been precisely analyzed. Moreover, the allosteric mode of MPER during membrane fusion has not been demonstrated. On the other hand, investigators need to focus on the failure to induce anti-MPER bNAbs in relation to the prevalence of autoreactivity/polyreactivity, as shown by the reported MPER-specific bNAbs.

Meanwhile, we need to further probe the matter of protection relative to the sufficiency of antibody-dependent cell-mediated cytotoxicity (ADCC) antibodies and the broader protection of bNAbs. Indeed, although bNAbs are crucial to protect humans from HIV-1 infection, the role of non-neutralizing antibodies, especially ADCC antibodies, in protection has been revealed in some studies. As mentioned above, the protective effect of RV144 vaccine is related to ADCC antibodies, rather than neutralizing antibodies, implying that neutralizing antibodies may be not necessary. In another study, vaginal IgA with ADCC and transcytosis-blocking activity induced by gp41-engrafted virions were closely related to the protection of NHP from SHIV infection (Bomsel et al., 2011). Recently, Sun et al. (2016) designed an immunogen containing four tandem 10E8 epitope repeats that exhibits α-helical conformation and the key amino acids W and F, which can point toward different directions when the long peptide binds the plasma membrane, thereby strengthening the induction of antibodies capable of binding to the native conformation of MPER on the viral envelope. After immunizing New Zealand rabbits with the immunogen, the ADCC reporter gene was activated, suggesting the existence of ADCC activity. However, the evaluation standard for protective effect of ADCC antibody was absent, leading to past neglect in the design and evaluation of an MPER-based vaccine.

In a word, the design of the MPER-based vaccine is replete with complications that require further elucidation; therefore, we still have a long way to go before the bNAbs dilemma, as detailed in this review, is settled. MPER, a conserved target, does, however, remain of vital interest, but apart from inducing neutralizing antibodies, non-neutralizing, yet protective, antibodies might be a future direction. Moreover, similar to “cocktail therapy”, the induction of combinational protective antibodies targeting several regions, such as V1V2 and MPER, may be required to achieve the best protection. If this strategy is adopted, MPER is one target particularly worthy of consideration.
